# *In silico* analysis of protein toxin and bacteriocins from *Lactobacillus paracasei* SD1 genome and available online databases

**DOI:** 10.1371/journal.pone.0183548

**Published:** 2017-08-24

**Authors:** Komwit Surachat, Unitsa Sangket, Panchalika Deachamag, Wilaiwan Chotigeat

**Affiliations:** 1 Department of Molecular Biotechnology and Bioinformatics, Faculty of Science, Prince of Songkla University, Hat Yai, Songkhla 90112, Thailand; 2 Center for Genomics and Bioinformatics Research, Faculty of Science, Prince of Songkla University, Hat Yai, Songkhla 90112, Thailand; 3 Information and Communication Technology Programme, Faculty of Science, Prince of Songkla University, Hat Yai, Songkhla 90112, Thailand; INSERM U869, FRANCE

## Abstract

*Lactobacillus paracasei* SD1 is a potential probiotic strain due to its ability to survive several conditions in human dental cavities. To ascertain its safety for human use, we therefore performed a comprehensive bioinformatics analysis and characterization of the bacterial protein toxins produced by this strain. We report the complete genome of *Lactobacillus paracasei* SD1 and its comparison to other *Lactobacillus* genomes. Additionally, we identify and analyze its protein toxins and antimicrobial proteins using reliable online database resources and establish its phylogenetic relationship with other bacterial genomes. Our investigation suggests that this strain is safe for human use and contains several bacteriocins that confer health benefits to the host. An *in silico* analysis of protein-protein interactions between the target bacteriocins and the microbial proteins gtfB and luxS of *Streptococcus mutans* was performed and is discussed here.

## Introduction

The term ‘probiotics’ has been defined by a joint consultation of experts from the Food and Agricultural Organization of the United Nations and World Health Organization as “live microorganisms which, when administered in adequate amounts, confer a health benefit on the host” [1]. They have diverse key functions, such as activities against other microorganisms [2], modulation of the host immune system [3], and the prevention of carcinogenic processes [4, 5], allergic diseases [6] and oral diseases [7–9]. The use of probiotics has been proposed for the maintenance of microbial homeostasis in the oral cavity, gastrointestinal tract and vagina. In addition, especially in oral health applications, probiotics could beneficially alter the biofilm microbial composition [10] or stimulate the host immune response [11]. One of the organisms with great potential to inhibit the growth and persistence of detrimental resident bacteria in specific oral areas is *Lactobacilli* sp. [12–14].

For administration as human oral probiotics, various strains and species of *Lactobacillus* have been studied. *Lactobacillus rhamnosus* GG [15], for example, has been investigated in vitro and tested in vivo in the oral cavity. However, the results reported that permanent colonization of the oral cavity was not consistently achieved. It seems to occur only in some individual patients. Another study [16] investigated the probiotic activity of *L*. *reuteri* DSM 17938, *L*. *acidophilus* DDS-1, *L*. *rhamnosus* ATCC 53103, and *L*. *paracasei* B21060 and characterized their benefits in terms of their reduction of surface tension and emulsifying ability. Against test pathogens *Streptococcus mutans* ATCC 25175 and *Streptococcus oralis* ATCC 9811, *Lactobacilli* significantly inhibited adhesion and biofilm formation on titanium surfaces. Although researchers have performed and proposed many studies about the oral origin of antimicrobial proteins or bacteriocins produced by *Lactobacilli*, more knowledge about their safety is still required.

Recently, Rawee et al. [17–19] isolated *L*. *paracasei* SD1 from the human oral cavity, and the isolate significantly reduced populations of oral *S*. *mutans*, perhaps discovering a new probiotic that can control bacterial communities in the human mouth. The results of the in vitro research reported a protein with an approximate molecular weight of 25 kDa that exhibited a broad spectrum against oral pathogens. This result showed the possible application of this bacteriocin in the prevention and treatment of oral diseases. However, even though the antibacterial activity produced by *L*. *paracasei* SD1 may have already been explored and characterized, the whole genome sequence and related knowledge must be thoroughly investigated before it may be considered safe for human applications.

In this paper, we will present and discuss an *in silico* analysis of bacterial protein toxins and bacteriocins from *L*. *paracasei* SD1. This study aims to sequence and assemble the whole genome, analyze all possible protein toxins, and identify all antimicrobial proteins or bacteriocins in the genome and plasmids. The workflows for the identification of protein toxins and bacteriocins were put together from available online databases, and these identifications are reported here. Many interesting bacteriocins were identified in the genome and plasmid of SD1: Carnocin-CP52, Enterocin X*β*, Gassericin A, LSEI_2163 and LSEI_2386. Additionally, the list of identified toxins in the SD1 genome was reported, and the safety of their use in dairy products is discussed. To show the binding potential and discuss the mechanism inhibiting the growth of *S*. *mutans*, we simulated *in silico* protein-protein interactions between the bacteriocins and the gtfB and luxS membrane proteins of *S*. *mutans*, which are involved in biofilm formation. This study could confirm the safety and benefits of *L*. *paracasei* SD1 in probiotic products due to its inhibition of other bacterial strains.

## Materials and methods

### Isolation and purification of genomic DNA

*L*. *paracasei* SD1 was donated by Prof. Dr. Rawee Teanpaisan of the Common Oral Diseases and Epidemiology Research Center, Faculty of Dentistry, Prince of Songkla University. DNA extraction was performed with DNeasy extraction kit (QIAGEN, Hilden, Germany) following the manufacturer’s instructions. Briefly, the bacterial cell pellet was resuspended in 180 *μ*L of enzymatic lysis buffer and incubated for 30 minutes at 37°C and 25 *μ*L of proteinase K 200 *μ*l Buffer AL was then added and mixed before incubating at 56°C for 30 min. Then, 200 *μ*L of ethanol was added to the DNA sample and centrifuged through the DNeasy Mini spin column at 610 g for 1 minute. After that, DNA was washed with 500 *μ*L of Buffer AW2 and then eluted with buffer AE. Finally, the DNA concentration in the eluate was measured by a spectrophotometer at 260 nm. The ratio of the absorbance at 260 nm and 280 nm (A260/A280) provided an estimate of the purity of DNA by agarose gel electrophoresis.

### Genome sequencing

The *L*. *paracasei* SD1 genome (Accession no. SRP091927) was sequenced using PacBio P6C4 chemistry on a PacBio RSII sequencer (Pacific Biosciences, California, USA). One single-molecule real-time (SMRT) cell was used for sequencing, yielding 150,292 subreads with an average read length of approximately 5 kbp. The hierarchical genome assembly process (HGAP) method based on the SMRT Analysis package v2.3.0 in the SMRT Portal was used for de novo assembly. The following assembly parameters were set: minimum subread length at 500 bp, minimum read score at 0.80 and length cutoff at 5,000 bp. There were 74,620,028 pre-assembled bases with 22,624 pre-assembled reads. As a result, the assembly yielded six contigs with with the largest contig being approximately 2.84 Mbp. The Gepard visualization tool [20] was used to evaluate the assembly by drawing the genome dot plot. Finally, Circlator [21], a tool for automated circularization of genome assemblies, was used to concatenate all the contigs into one closed circular chromosome and two circular plasmids.

### Genome and plasmid annotation

Initial automated gene calling was performed using Glimmer 3 [22] and Genemark [23–25]. Functional annotation was achieved using RAST (Rapid Annotation using Subsystem Technology) [26, 27], tRNA was predicted by tRNAscan-SE 1.21 [28–31] and rRNA genes were predicted by RNAmmer 1.2. The CDS, genes and related regions were annotated by mapping the plasmids to a plasmid database, which was downloaded from NCBI on 1-07-2016. All the predicted proteins were searched (BLASTP) against the NCBI non-redundant (nr) protein database. Prophage regions were predicted using the PHAge Search Tool (PHAST) web server [32], and regions of clustered regularly interspaced short palindromic repeats (CRISPR) were searched using the CRISPRFinder server [33].

### Genome comparisons

*L*. *paracasei* SD1 was first searched with the microbial nucleotide BLAST to find the closest organism for comparison. *L*. *paracasei* JCM 8130 (NC_008526), *L*. *casei* ATCC 334 (NC_008526) and *L*. *paracasei* KL1 (NZ_CP013921) were the top three blast hits with approximately 99% identity and very significant E-values. Based on the BLASTX comparison, the similarity of SD1 with its three closest relatives was analyzed and visualized using the CGView Server [34] by setting the expect value cutoff to 0.00001 and the identity cutoff to 30%.

### Phylogenetic analysis

16S rRNA sequences (approximately 1,540 bp) were retrieved from the following 32 species: *L*. *johnsonii* NCC 533, *L*. *johnsonii* FI9785, *L*. *johnsonii* N6.2, *L*. *johnsonii* BS15, *L*. *acidophilus* La-14, *L*. *acidophilus* NCFM, *L*. *acidophilus* FSI4, *L*. *reuteri* DSM 20016, *L*. *reuteri* SD2112, *L*. *reuteri* ZLR003, *L*. *reuteri* I49, *L*. *plantarum* DF, *L*. *plantarum* B21, *L*. *plantarum* WCFS1, *L*. *plantarum* KP, *L*. *salivarius* st. Ren, *L*. *salivarius* JCM 1046, *L*. *salivarius* CECT 5713, *L*. *salivarius* UCC118, *L*. *paracasei* KL1, *L*. *paracasei* JCM 8130, *L*. *paracasei* N115, *L*. *paracasei* L9, *L*. *casei* Zhang, *L*. *casei* LOCK919, *L*. *casei* BD-II, *L*. *casei* W56, *L*. *rhamnosus* LOCK908, *L*. *rhamnosus* BFE5264, *L*. *rhamnosus* ATCC 8530, *L*. *rhamnosus* ATCC 53103 and *L*. *lactis* KF147. A multiple alignment of the 16S rRNA nucleotides was generated using MUSCLE [35]. The phylogenetic tree was constructed by Geneious 9.1.2 [36, 37] using the neighbor-joining method with a bootstrap value of 1,000.

### Genome and plasmid visualization

The circular representation of the chromosome of *L*. *paracasei* SD1 was produced using the CGView Server V 1.0 [34]. The subsystem mapping was produced by RAST version 2.0 [26, 27]. In addition, the circular representation of the plasmids was drawn using Geneious 9.1.2 [36, 37].

### Protein toxin identification

The workflow was constructed with two main processes: a preliminary identification and the local blast and sequence alignment. The preliminary identification was implemented to search related genes and proteins using BLASTN in the VFDB database [38–41] and BLASTX in the DBETH database [42], respectively. The preliminary result list was generated individually. We then further analyzed and investigated the results by searching the NCBI and the UniProt databases and gathering a set of similar genes and proteins in *Lactobacilli* organisms to create our own local databases. Using BLASTN with the NCBI nucleotide sequence and BLASTX with the UniProt protein sequence, we set the organism to *Lactobacillus* and E-value to 10^−4^ to create our own local databases. Finally, the result lists were compiled by searching the preliminary results with the created databases and aligning sequences individually with the references. The final result was the confirmed list of genes and protein toxins in *L*. *paracasei* SD1. The workflow of protein toxin identification is given in S1 Fig.

### Antibiotic-resistant protein identification

The presence of antibiotic-resistance genes in *L*. *paracasei* SD1 was analyzed by using hmmscan to the proteomes of the chromosome and plasmids against the Resfams database [43]. The possibility of horizontal transfer of antibiotic-resistance genes to other commensals was then identified by searching against the ICEberg database [44].

### Bacteriocin identification

**Fig 1:**
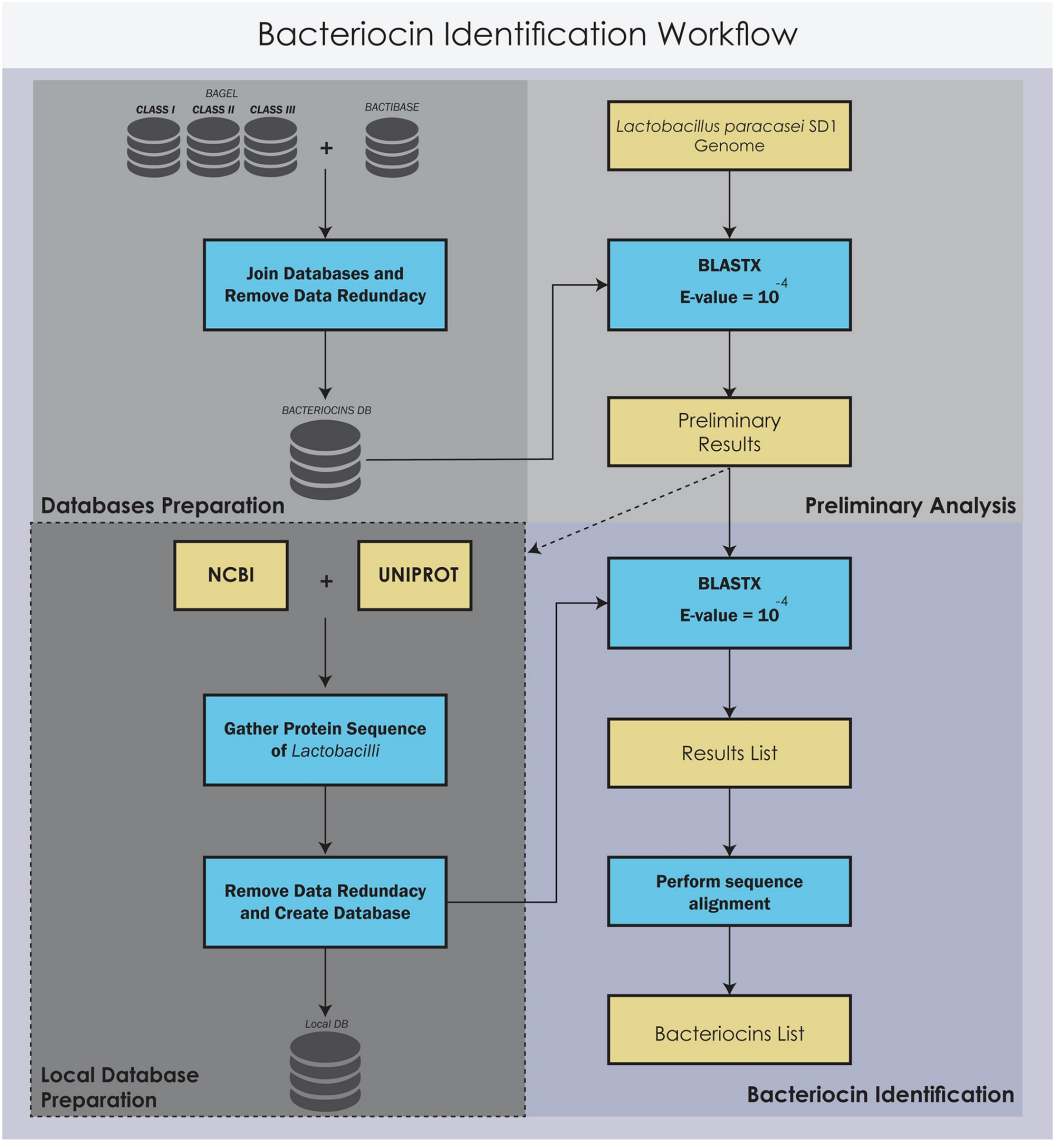
The bacteriocin identification workflows. There were four processes in the workflow: database preparation, preliminary analysis, local database preparation, and bacteriocin identification.

The first step of the bacteriocin identification workflow was creating a *BACTERIOCINS* local database by merging the *BAGEL* (class I, II and III) [45] and *BACTIBASE* databases [46, 47]. The nucleotide sequence of *L*. *paracasei* SD1 was first screened with the *BACTERIOCINS* database using BLASTX. The preliminary screening result was then generated and used as the target of a search for similar protein sequences from *Lactobacilli* in the NCBI and UniProt databases. The local database was then created after removing data redundancy in the database. Finally, the preliminary result from the first blast was then searched again against the newly created database using BLASTX with the E-value set to 10^−4^ and an identity cutoff at 30%. The final result was then aligned to find the conserved sequence and confirm it as a bacteriocin in *L*. *paracasei* SD1. The workflow is given in Fig 1.

### Protein-protein interaction analysis

The 3D structure of three bacteriocins, namely, LSEI_2163, LSEI_2386, and Gassericin A of *L*. *paracasei* SD1, were predicted using the RaptorX server [48–51], I-TASSER server [52–56] and QUARK server [57]. The predicted models were then evaluated using RAMPAGE [58] and PROCHECK [59]. The predicted model of LSEI_2163 came from the RaptorX server, whereas the other two models were from the I-TASSER server due to their higher scores and the absence of residues in allowed regions and outlier regions. In addition, the sequences of gtfB and luxS of *S*. *mutans* were obtained from the UniProt database with primary accession numbers Q8DVK8 and P08987, respectively.

The 3D structures of luxS and gtfB were then modeled by SWISS-MODEL [60–64]. The templates for luxS and gtfB were S-ribosylhomocysteine lyase from *S*. *mutans* strain ATCC 700610/UA159 (SMTL id: 4xch.1.A, X-RAY DIFFRACTION 2.20 Å, residue 12–157) with 82.17% sequence identity and the crystal structure of Glucosyltransferase-I from *S*. *mutans* strain ATCC 700610/UA159 (SMTL id: 3tto.1.A, X-RAY DIFFRACTION 3.30 Å, residue 159–1058) with 48.95% sequence identity, respectively Then, protein-protein docking was performed by the PIPER program [65] on the ClusPro 2.0 server [66–68]. The model with the largest cluster and the lowest docking score of the balance mode was selected. Afterwards, the selected model was elucidated by the PyMOL program [69]. The determined values included scores for balanced, electrostatic, hydrophobic, and Van der Waals interactions.

## Results and discussion

### Genome features of *L*. *paracasei* SD1

Whole-genome sequencing was processed with the PacBio single-molecule real-time (SMRT) sequencing system to determine the genome sequence of *L*. *paracasei* SD1. De novo assembly generated five contigs using the HGAP v3.0 from PacBio reads obtained using P6C4 chemistry. We then further assembled and verified the finished single complete genome. The genome of *L*. *paracasei* SD1 composes of one circular chromosome (2,995,875 bp) and two circular plasmids designated, as pSD1-1 (11,281 bp) and pSD1-2 (10,902 bp). The general genomic features and information are given in Table 1.

**Table 1:** Genome and plasmid features and information for *Lactobacillus paracasei* SD1.

	SD1 genome	pSD1-1	pSD1-2
Sequencing technology	PacBio P6C4		
BioProject number	PRJNA349248		
BioSample number	SAMN05929041		
Submission number	SRP091927		
Size (in bp)	2,995,875	11,281	10,902
GC content (%)	46.6	42.5	40.2
CDS	2984	15	10
Hypothetical protein	962	11	8
Subsystems	338	-	-
tRNA	61	-	-
rRNA	15	-	-

Four intact prophages were predicted in chromosome *ϕ*SD1-1 (681–709 kb), *ϕ*SD1-2 (822–864 kb), *ϕ*SD1-3 (1,828–1,876 kb) and *ϕ*SD1-4 (1,939–1,993 kb). No prophage was identified in the plasmids. Most of the identified prophage CDS were hypothetical proteins. The prophages are known as the inactive forms of the bacteriophage and are commonly found in *Lactobacillus* organisms. In addition, we also explored some *Lactobacillus* organisms and identified prophage regions to compare with *L*. *paracasei* SD1. We investigated the genomes of *L*. *paracasei* JCM 8130, *L*. *paracasei* strain KL1, *L*. *casei* ATCC 334, *L*. *paracasei* 8700:2, *L*. *casei* W56, *L*. *casei* BD-II chromosome, *L*. *casei* LOCK919, *L*. *brevis* ATCC 367, *L*. *fermentum* IFO 3956, *L*. *plantarum* WCFS1, *L*. *reuteri* DSM 20016, *L*. *rhamnosus* GG, and *L*. *salivarius* UCC118. As a result, we identified predicted prophages in every target genome. The prophage contents of *Lactobacilli* were similar, and they shared many CDS in the chromosome.

Additionally, we used the IslandViewer 3 application [70] to identify genomic islands (GIs) in *L*. *paracasei* SD1. Seventeen GIs were predicted with an average length of 9.2 kbp, corresponding to a total sequence 156 kbp or 5.22% of the genome. This externally acquired DNA percentage is lower than that of the reference genome, *L*. *casei* ATCC 334, with 8.76%, but it is higher than those of other members of the *L*. *paracasei* group, which ranged from 1.6% in *L*. *paracasei* JCM 8130 to 0.84% in *L*. *paracasei* KL1. Multiple mobile genetic elements (MGEs), including prophages and transposons, were identified in the GI elements. Most of the MGEs found in the genome contained similarities to integrative and conjugative elements (ICEs), which can be integrated into or excised from the host chromosome. Normally, ICEs provide increased adaptation to the host, through genetic improvements to phage resistance and metal transport, for instance. Genes similar to transmembrane proteins and phage-related proteins were identified in the genome. We suggest that these genes are related to increasing the incorporation and stability of this bacterial strain in the human oral cavity, which thus improves its local fitness.

**Fig 2:**
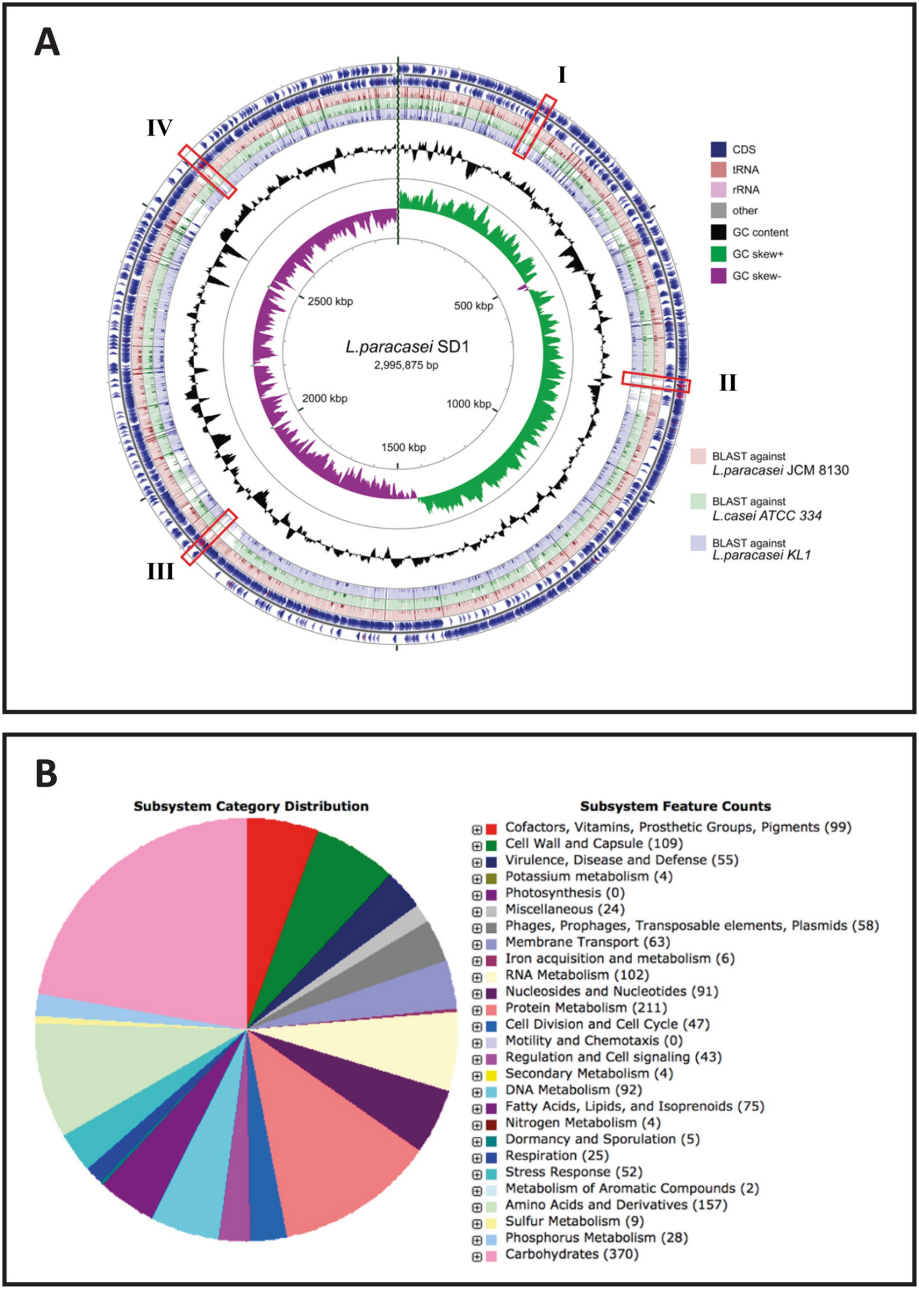
(A) Circular representation of the SD1 chromosome and (B) functional distribution of the SD1 coding genes based on SEED subsystems. (A) The outermost circle and the second circle show the positions of the CDS in clockwise and counterclockwise directions, respectively. Protein-coding genes, tRNA genes and rRNA genes are highlighted in different colors. The third, fourth, and fifth circles from the outside represent BLASTX comparisons with *L*. *paracasei* JCM 8130, *L*. *casei* ATCC 334 and *L*. *paracasei* KL1, respectively. The innermost and second inner circle illustrate the GC skew and the GC content, respectively. GC skew+ and GC skew- are distinguished by different colors. The red box represents the unique region that appears only in SD1. (B) Approximately 41% of protein-coding genes were assigned SEED functional categories.

In addition, no CRISPR loci were identified in *L*. *paracasei* SD1. NCBI microbial nucleotide BLAST was used to evaluate its similarity with other microbial genomes. The *L*. *paracasei* SD1 genome was searched against all completed genomes in the NCBI databases. The closest microbial organism was *L*. *paracasei* JCM 8130, followed by *L*. *casei* ATCC 334 and *L*. *paracasei* KL1. The identity of the top three closest genomes was around 99%. The similarity based on BLASTX comparison with its three close relatives was analyzed and visualized as shown in Fig 2. The red boxes highlight several unique regions in *L*. *paracasei* SD1 (region I: from ≈ 28° to 32°, region II: from ≈ 88° to 92°, region III: from ≈ 224° to 227° and region IV: from ≈ 308° to 312°). Several CDS, including transposase proteins, transcriptional regulators, putative membrane proteins, FPXTG cell wall anchor family proteins, and alpha-galactosidase, were inserted in those regions (S1 Table), which may have affected the strain’s adaptation to specific ecological niches during its evolution.

**Fig 3:**
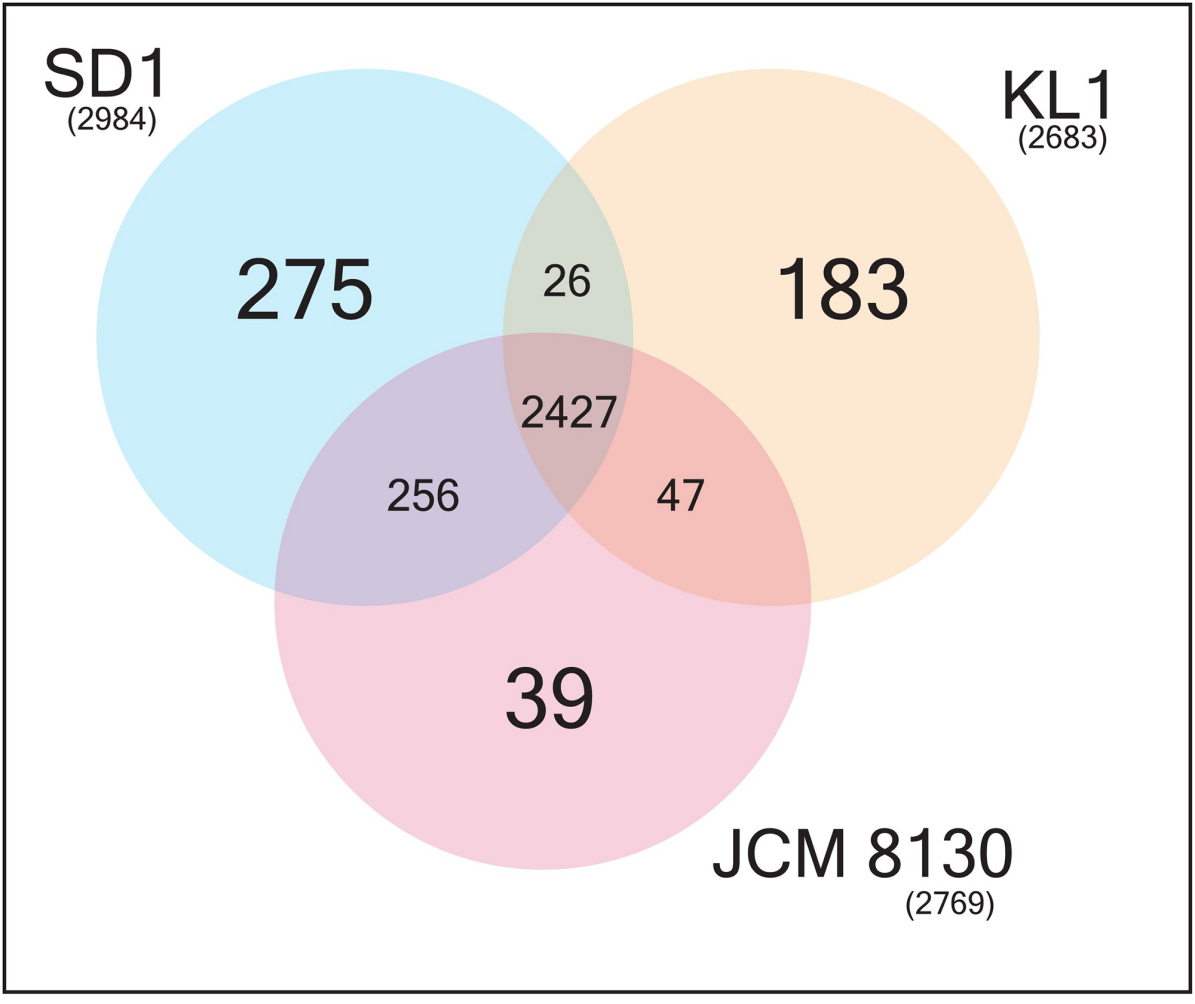
Core, dispensable and unique sets of proteins. Venn diagram of ortholog group distribution in *L*. *paracasei* SD1, *L*. *paracasei* JCM 8130, and *L*. *paracasei* KL1.

Orthologous proteins among the closest strains of *L*. *paracasei* SD1 were obtained using the reciprocal all versus all BLAST. The analysis revealed that 2427 proteins formed the core set of proteins among the three strains. Orthology charts were plotted from the orthologous proteins as shown in Fig 3. *L*. *paracasei* SD1 shared 2,683 orthologous pairs with *L*. *paracasei* JCM 8130 and 2,453 with *L*. *paracasei* KL1. The remaining proteins that contained no orthologous pairs were then extracted from the proteomes of *L*. *paracasei* SD1 (275 proteins) and subjected to BLASTP against the NR database. Out of 275 proteins in *L*. *paracasei* SD1, only eighteen (37–54 residues) did not match any sequence in the database and 168 proteins were annotated as hypothetical proteins. The rest of the proteins had homology to *L*. *paracasei* and *L*. *casei* proteins at > 85% identity and < 10^−5^ E-value.

### Plasmid features of *L*. *paracasei* SD1

Two circular plasmids, pSD1-1 (11,281 bp) and pSD1-2 (10,902 bp) were identified during the assembly process. General information on pSD1-1 and pSD1-2 are given in Table 1. The sizes of these two plasmids are slightly different. The first plasmid, pSD1-1, is comprised of various regions from different species/subspecies of *L*. *paracasei*, *L*. *casei* and *L*. *gallinarum*, which indicates the evolution among them. There were 15 CDS in this plasmid, and 8 of them were annotated as hypothetical proteins. The other CDS comprised 1 ABC transporter, 1 cell division protein FtsX, 2 mobilization proteins, 2 plasmid replication proteins (RepB), and 1 Gassericin A. Interestingly, Mob plasmid mobilization proteins were identified in this plasmid and are probably required for relaxation complex formation and plasmid mobilization by conjugative plasmids. Another fascinating characteristic was the presence of a bacteriocin gene, Gassericin A, that contains antibacterial activity against a number of gram-positive food-borne pathogenic bacteria [71]. This mechanism may play an important role in inhibiting other bacterial activities in human.

The other plasmid, pSD1-2, shared many proteins with various plasmids and genomes of the bacteriocin-producing strain of *Lactobacilli*, such as plasmid pLBPC-2 of *L*. *paracasei* subsp. paracasei JCM 8130, the plasmid unnamed1 of *L*. *paracasei* strain CAUH35, plasmid pW56 of *L*. *casei* W56, and plasmid pBD-II of *L*. *casei* BD-II. These strains also produce many bacteriocins, but there was no sign of antimicrobial proteins in these plasmids similar to pSD1-2 of SD1. The content of pSD1-2 comprised 10 CDS, which were annotated to 4 hypothetical proteins, 1 serine protease, peptidoglycan-binding protein, 2 RepB, 1 SMI1/KNR4 family protein, and 1 protein that was not found in any of the available databases. In the case of SMI1/KNR4, this protein is found in many species of *Lactobacillus*, *Streptococcus*, *Geobacillus*, *Listeria*, *Bacillus*, and others. Originally, SMI1/KNR4 was found in a yeast cell wall. It plays a regulatory role in chitin deposition and cell wall assembly, and it connects to other pathways for cell proliferation. We can infer that SMI1/KNR4 may have transferred during the evolution process while remaining functional in the plasmid of this bacterial strain. The features of both plasmids, pSD1-1 and pSD1-2, are illustrated in S2 Fig.

### 16S rRNA phylogenetic analysis of *L*. *paracasei* SD1 and other sequenced *Lactobacilli*

**Fig 4:**
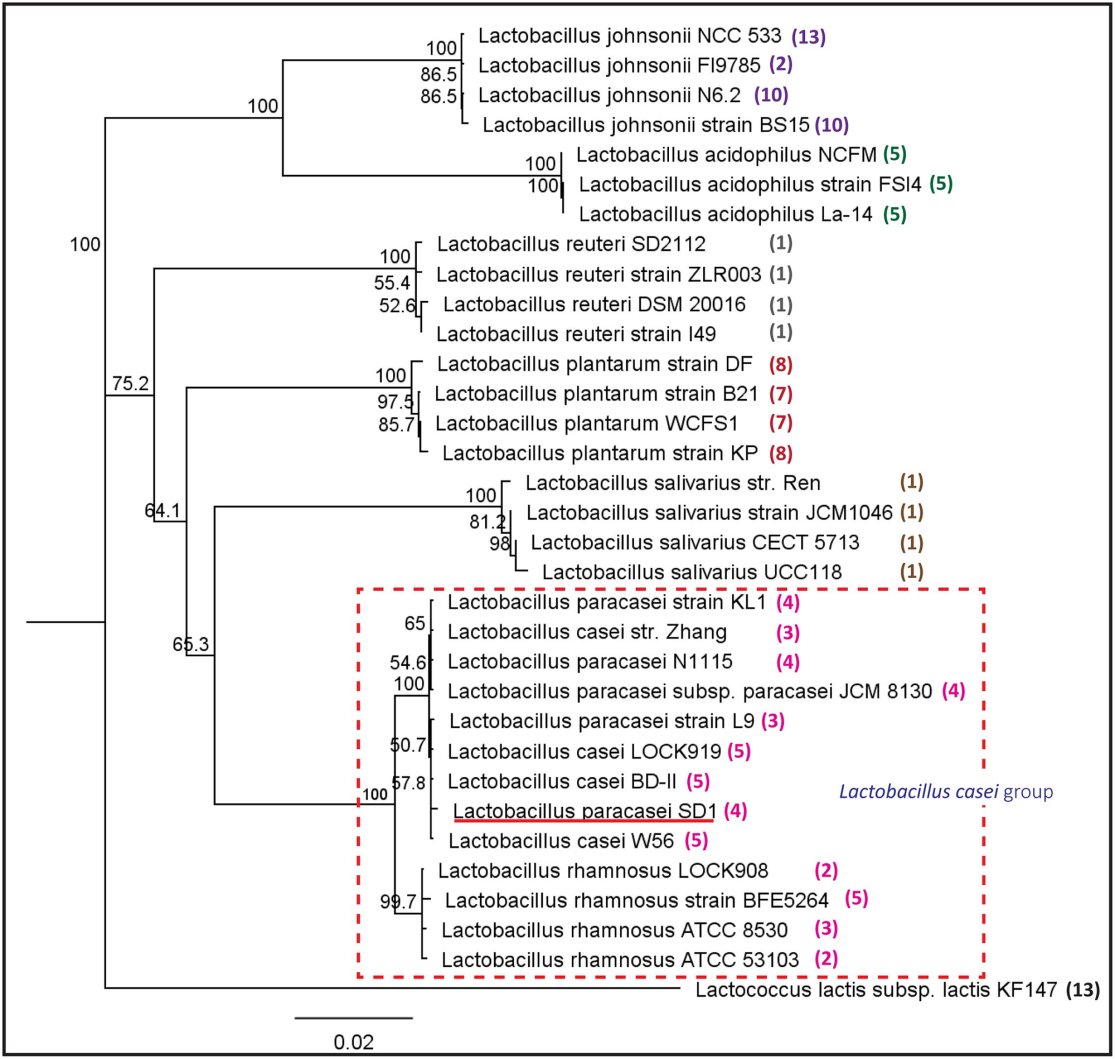
Phylogenetic tree based on the neighbor-joining method of 16S rRNA gene sequences. The analysis involved 34 sequences of *Lactobacillus* species. The sequences were retrieved from 3 *L*. *acidophilus*, 4 *L*. *casei*, 4 *L*. *johnsonii*, 4 *L*. *paracasei*, 4 *L*. *plantarum*, 4 *L*. *reuteri*, 4 *L*. *rhamnosus*, 4 *L*. *salivarius*, and 1 *Lactococcus lactis*. *L*. *lactis* KF147 was set as the outgroup. The tree was created using a neighbor-joining method and numbers at branch points are bootstrap values (based on 1000 samplings expressed in percentages). The scale bar represents an evolutionary distance. The tree has been arbitrarily rooted. The number of bacteriocins produced by each strain is shown in parentheses.

16S rRNA sequences (approximately 1,540 bp) were collected from *Lactobacilli* genomes (3 *L*. *acidophilus*, 4 *L*. *casei*, 4 *L*. *johnsonii*, 4 *L*. *paracasei*, 4 *L*. *plantarum*, 4 *L*. *reuteri*, 4 *L*. *rhamnosus*, 4 *L*. *salivarius*, and 1 *Lactococcus lactis*). There are many species of *Lactobacilli* that have more than one copy of ribosomal RNA in their genome; therefore, we used representative consensus sequences [72, 73] from multiple sequence alignment. Then, we constructed the phylogenetic relationships between *L*. *paracasei* SD1 and the others based on thirty-three 16S rRNA sequences, setting *L*. *lactis* KF147 as the outgroup. Additionally, we also identified the amount of bacteriocins produced by each strain in the chromosome using the *BAGEL* database. The result in Fig 4 shows that *L*. *paracasei* SD1 was placed in the casei group, which formed a distinct clade from *L*. *rhamnosus*. SD1 produced 4 bacteriocins in the genome, which was almost the same amount as the others in this clade. In addition, the produced bacteriocins shared among this group were the same protein, i.e., LSEI_2163, LSEI_2386 and Enterocin X*β*. This result could reveal how SD1 is phylogenetically close to the other members of this group.

### Protein toxin report

There were 9 different protein toxins from 15 locations in the preliminary screening results from blasting the DBETH database [42] and just 6 records containing E-values less than 0.0001. We eliminated some results that contained E-values higher than 1e-4. The remaining records were 2 Hemolysin (1 from *Vibrio cholerae* MO10 and 1 from *Vibrio cholerae* CT 5369-93), 2 Hemolysin-III (1 from *Bacillus cereus* and 1 from *Clostridium tetani*), lepB and Phospholipase D. Thus, we further investigated the preliminary results by gathering information about the identified protein toxins in *Lactobacilli* from NCBI and UniProt and creating a local database. The blast results against the local databases confirmed that Phospholipase D was not found in any part of *L*. *paracasei* SD1. However, we identified a protein toxin in the Hemolysin family with a very low E-value and 100 percent identity with Hemolysin and *Hemolysin-III* in 2 different locations. These two proteins are also commonly found in many close organisms, such as *L*. *casei*, *L*. *paracasei*, *L*. *rhamnosus*, *L*. *zeae*, and *L*. *saniviri*. Unsurprisingly, there are many reports about Hemolysin and its safety in *Lactobacilli* [74, 75]. These reports suggested that *Lactobacilli* can be included in probiotic products without any danger to the host organism [1, 74–77].

The signal peptidase lepB was also investigated using the local database. The results contained very low E-values and very high percent identity (over 99%). This points to the presence of only three protein toxins in *L*. *paracasei* SD1, as shown in Table 2. The complete sequences of the three identified toxins are also given in S2 Table. We also searched the bacterial gene toxin in the VFDB database. No significant similarity was found with any of the genes.

**Table 2:** Protein toxins identified in the SD1 genome and plasmid using BLASTX against DBETH database.

Protein Name	Bit-Score	Identity	Length	Mismatches	E-value	Location	Frame
Hemolysin-III	118.24	35.29	204	132	8.00E-28	1,386,627–1,387,238	-2
Hemolysin	77.41	23.4	265	184	5.00E-14	1,773,619–1,774,407	-2
lepB	58.92	25.73	206	95	5.00E-08	252,436–252,933	-1

Hemolysis is a common virulence factor in microorganisms. It can facilitate iron availability and cause anemia and edema in the host [76]. These microorganisms need iron as a cofactor for several enzymes [78]. *Lactobacilli* can grow normally without iron, which is an ecological advantage in the natural environment, where they compete with pathogenic bacteria. That advantage could imply that the Hemolysin protein family found in *Lactobacilli* does not cause the lysis of human erythrocytes [77, 79, 80].

### Antibiotics-resistant protein report

Presently, antibiotic-resistance is a major problem in treating bacterial infections. Antibiotic resistance can naturally occur or can be achieved by the overuse and misuse of antibiotics. Bacteria or other microbes can develop resistance to antibiotics by mutating existing genes or by acquiring new genes from other strains or species by genetic mechanisms, such as horizontal gene transfer through plasmids or transposons. Several species of *Lactobacillus* have been commercially used as probiotics. Thus, a safety concern due to the possibility of transferring antibiotic resistance via their plasmids or transposons should be identified and discussed.

We identified the presence of antibiotics-resistant proteins in both the chromosome and plasmids of *L*. *paracasei* SD1. The genome of SD1 was then subjected to hmmscan against the Resfams database [43]. There was no sign of the presence of any antibiotic-resistant proteins in plasmid pSD1-2. However, 3 Resfams domains were identified in plasmid pSD1-1. These domains had the same antibiotic mechanism, which is ABC transporters. ABC transporters are present in all cells of all organisms and use ATP binding energy to transport substrates across cell membranes. In the chromosome, 80 Resfams domains were identified, which can be classified by their antibiotic mechanisms into 14 groups (S3 Table). Beta-Lactamase, glycopeptide resistance and gene modulating resistance were the top three groups found in SD1. We then analyzed the possibility of horizontal transfer of the identified proteins to other commensals by searching the ICEberg database [44]. ICEberg is a database for integrative and conjugative elements (ICEs) found in both gram-positive and gram-negative bacteria. ICEs are self-transmissible mobile genetic elements that can be integrated into or excised from the host chromosome. Thus, they can promote their own mobilization and horizontal transfer of virulence determinants, antibiotic-resistance genes and other bacterial traits. We then used plasmid pSD1-1 as a search target in the ICEberg database using the BLASTN program by with the expect value cutoff set to 1e-4. No matches came from plasmid pSD1-1. Thus, we suggested that the transfer of any antibiotic-resistance genes from the plasmid should not occur at this stage because its lack of ICEs and relevant elements. In addition, no large mobile genetic elements were identified in the chromosome. However, antibiotic resistance may be transferred via plasmid transfer. There were many short fragments matches to the database with small lengths (between 47–96 bp). Their percentage of query sequence coverage was too small compared to the target sequences. Consequently, we suggested that SD1 could not transfer any antibiotic-resistance genes to other commensals.

### Bacteriocin report

We identified four different bacteriocins in five different locations in the genome and four different bacteriocins in the same location as plasmid pSD1-1 as shown in Table 3. After further investigation with the locally created bacteriocin database of *Lactobacilli*, we suggest that the *L*. *paracasei* SD1 genome has a number of bacteriocins: LSEI_2163, LSEI_2386, Carnocin-CP52 immunity protein and Enterocin X*β*. Additionally, in the *L*. *paracasei* SD1 plasmid, we identified a bacteriocin of the Gassericin family. The sequences of all bacteriocins and their molecular weights are given in S4 Table.

**Table 3:** Bacteriocins identified in the SD1 genome and plasmid by blasts against BACTIBASE and BAGEL databases.

Protein Name	Bit-Score	Identity	Alignment Length	Mismatches	E-value	Location	Frame
LSEI_2386	95.9	100	45	0	6.00E-23	2,423,659 2,423,793	-1
LSEI_2163	84.34	100	39	0	6.00E-19	2,207,159 2,207,275	-3
Carnocin-CP52 immunity protein	65.47	31.82	110	74	7.00E-12	2,432,966 2,433,292	-3
Enterocin X*β*	46.60	50.98	51	23	4.00e-13	2,428,795 2,428,947	-1
	41.20	64	25	9	4.00E-04	2,431,600 2,431,674	-1
Butyrivibriocin AR10	72.40	55.0	60	27	5.05E-18	5,932 6,111	-2
Pentocin	59.31	40.7	81	44	4.10E-13	5,926 6,168	-2
Gassericin A	49.68	37.8	74	42	5.41E-10	5,932 6,153	-2
Acidocin B	48.13	50.0	44	22	2.06E-09	5,932 6,063	-2

Roughly, 30–90% of bacteria can produce at least one bacteriocin [81] which could imply that there are many undiscovered bacteriocins in both producing and non-producing strains of bacteria. Based on published knowledge, we identified at least five bacteriocins in this strain, as mentioned above. Two of them, LSEI_2163 and LSEI_2386, were putative pheromone peptides that have significant bacteriocin activity against several *Listeria* species [82], which could make this bacterium useful in medical applications. Enterocin X*β* is a class IIb bacteriocin that was originally found in *E*. *faecium* KU-B5 [83]. However, no research has reported Enterocin X*β* present in *Lactobacillus*. Only *Enterocin A* protein has been reported in the *Lactobacillus* genus according to study [84].

From our identification results (51% identity, 80.46% coverage, 23 bases out of 51 were mismatched, and an E-value approximately 4e-13), we could not exactly confirm the presence of Enterocin X*β* in the genome. We identified a related result with 100% identity for bacteriocin leader domain-containing protein, which is found in many strains of *Lactobacillus casei* and *paracasei*. However, we suggest that our proposed strain may contain bacteriocin-like proteins and still need to explore their mechanisms and antibacterial activity. In addition, we cannot definitely confirm that the Carnocin-CP52-like protein found in this genome was the same as the bacteriocin in the bacteriocin databases. It seemed to contain high confidence, with an of E-value approximately 3e-20, but its identity and coverage percentages were 32% and 98.20%, respectively. There were 74 mismatched bases out of 110.

**Fig 5:**
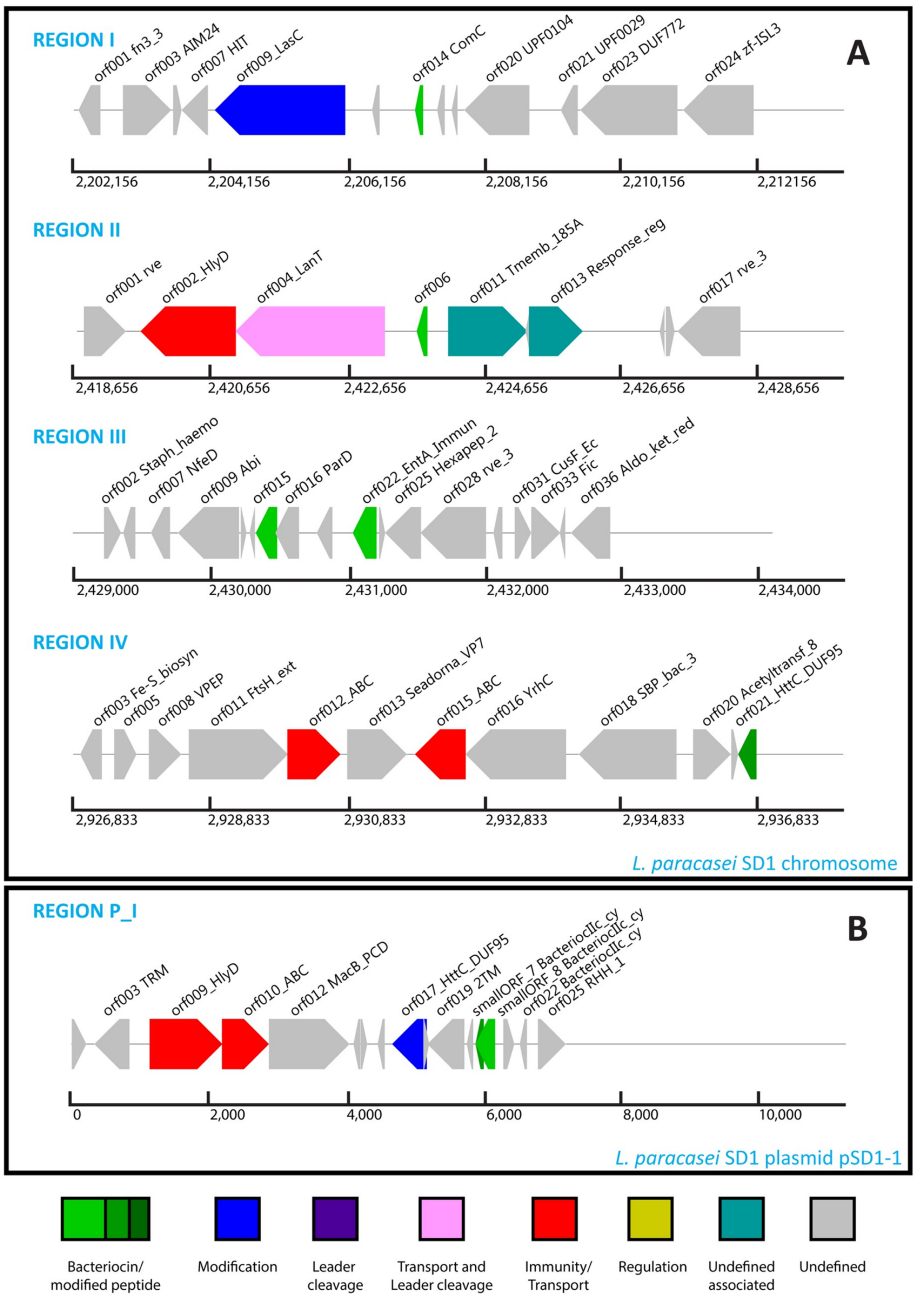
Gene clusters of the SD1 chromosome and plasmid. (A) Region I-IV represents gene clusters identified in the chromosome. (B) Region P_I represents gene clusters identified in the plasmid pSD1-1.

Further investigation was then conducted with BAGEL3, a web-based bacteriocin mining tool. BAGEL3 combines direct and indirect mining by looking at context genes. Therefore, it can explore bacteriocin-encoding genes or other bacterial ribosomally synthesized and post-translationally-modified peptides (RiPPPs) in the target genetic data. The result from BAGEL3 showed that bacteriocins were identified in four regions of the chromosome, as shown in Fig 5A. Other biosynthetic genes were present in regions I, II and IV. In region III, only two bacteriocin genes were identified. Region I contained a 9,944-bp area of interest comprised of two genes. This cluster contained a glutamine amidotransferase domain and a putative class II bacteriocin gene (LSEI_2163). Region II was found to contain a 9,944-bp class II bacteriocin cluster containing five genes, including one immunity/transport, one transport and leader cleavage, one bacteriocin and two undefined associated sites. An immunity/transport site that was found in this cluster was significantly similar to the bacteriocin ABC transporter, plnH. A transport and leader cleavage site, Bacteriocin ABC exporter (Pep2E family), was identified in this cluster. This protein involves bacteriocin transmembrane transporter activity, ATPase activity and is coupled to transmembrane movement of substances. The other genes were comprised of a putative class II bacteriocin gene (LSEI_2386), a response regulator (plnD) and one protein belonging to transmembrane fragile-X-F protein domain. Region III contained only two class II bacteriocin genes, Carnocin-CP52 immunity protein and Enterocin X*β*. There no signs of any biosynthetic genes. With the lack of a bacteriocin-associated domain in the cluster, we can only suggest that this strain may contain some immunity-like proteins, which have been submitted in the UniProt and NCBI databases under many names: possibly putative carnobacteriocin-B2 immunity protein, bacteriocin immunity protein, prebacteriocin, and others.

Surprisingly, we did not find any bacteriocins in region IV using BLASTX against the bacteriocin database. In contrast, BAGEL3 can predict a bacteriocin from this cluster. There were two immunity/transport domains, including a Type II/IV secretion system protein and a Nod factor export ATP-binding protein I. A head to tail cyclized peptide identified in this region did not match any record in the database. The sequence was then used to BLASTP search the UniProt database. The hit result with 100 percent identity was a membrane protein that was reported in many organisms of the *Lactobacillus* species. This protein belongs to Stage II sporulation protein M (SpoIIM), which is necessary for the forespore inside the mother-cell to be properly internalized through the breakdown of peptidoglycans trapped between the membranes of the septum separating the forespore and the mother-cell. SpoIIM was originally reported in *Bacillus subtilis*, where it was responsible for localization to the septal membrane.

In addition to the bacteriocins identified in the genome, there was also 1 bacteriocin in plasmid pSD1-1, Gassericin A, as shown in Fig 5B. This cluster contained two immunity/transport domains including a YlqD protein and a Type II/IV secretion system protein, and one modification domain, NADH dehydrogenase. Gassericin A is a circular bacteriocin produced by lactic acid bacteria that was originally discovered in *L*. *gasseri*. Gassericin A inhibits the growth of a wild type of toxic *S*. *aureus* isolated from mastitis milk and breast organs [71, 85, 86]. Additionally, there was another submission of Gassericin A in the *L*. *paracasei* Lpp41 strain, which could be an important finding, since it can be a crucial tool in food preservation due to its high *pH* and temperature tolerance [85].

### Bacteriocin interaction

Previous reports have shown that *S*. *mutans* and *S*. *sobrinus* were inhibited by *L*. *paracasei* SD1 [18, 19], but the mechanism is still unknown. In this *in silico* study, protein interactions between identified bacteriocins and the gtfB and luxS proteins of *S*. *mutans* were performed. Three bacteriocins from *L*. *paracasei* SD1, namely, Gassericin A, LSEI_2386 and LSEI_2163, were selected to be paired and docked to the target proteins because previous studies reported [82, 87–90] that those bacteriocins exhibited antimicrobial activity and inhibited several bacteria in vitro.

The reported mode of action of Gassericin A was to permeate the membrane and cause bacteriolysis [91]. However, the experiment performed by Kawai et al. [92] showed that bacteriocin concentrations were higher than what was required for antimicrobial activity in vivo. In addition, it has been a controversial issue as to whether bacteriocins require a receptor molecule or not. There was some evidence that the antimicrobial activity of class II bacteriocins was the receptor mediated by recognition of specific proteins on the membrane of target cells [93]. According to a report [94], some species of *Lactobacillus* that may contain the Gassericin family have the ability to decrease the biofilm formation of clinically isolated *S*. *mutans*. We then perform an *in silico* survey of the binding any target protein on the membrane of *S*. *mutans*.

The binding of bacteriocin to gtfB and luxS is based on how bacteriocin can control other microorganisms with gtfB and luxS as examples. gtfB from *S*. *mutans* produces water-insoluble glucans that have the ability to adhere to smooth and aggregated bacterial cells and food debris: thus, dental plaque occurs. We showed that the binding between bacteriocins and gtfB supports the prevention of gtfB to induce plaque. In addition, luxS is a S-ribosyl homocysteine lyase that involves the synthesis of auto inducer Al-2, a signaling molecule for quorum sensing in response to population density. Bacteriocin bound to luxS may therefore interfere with signaling communication, and the density of microorganisms will be reduced.

**Fig 6:**
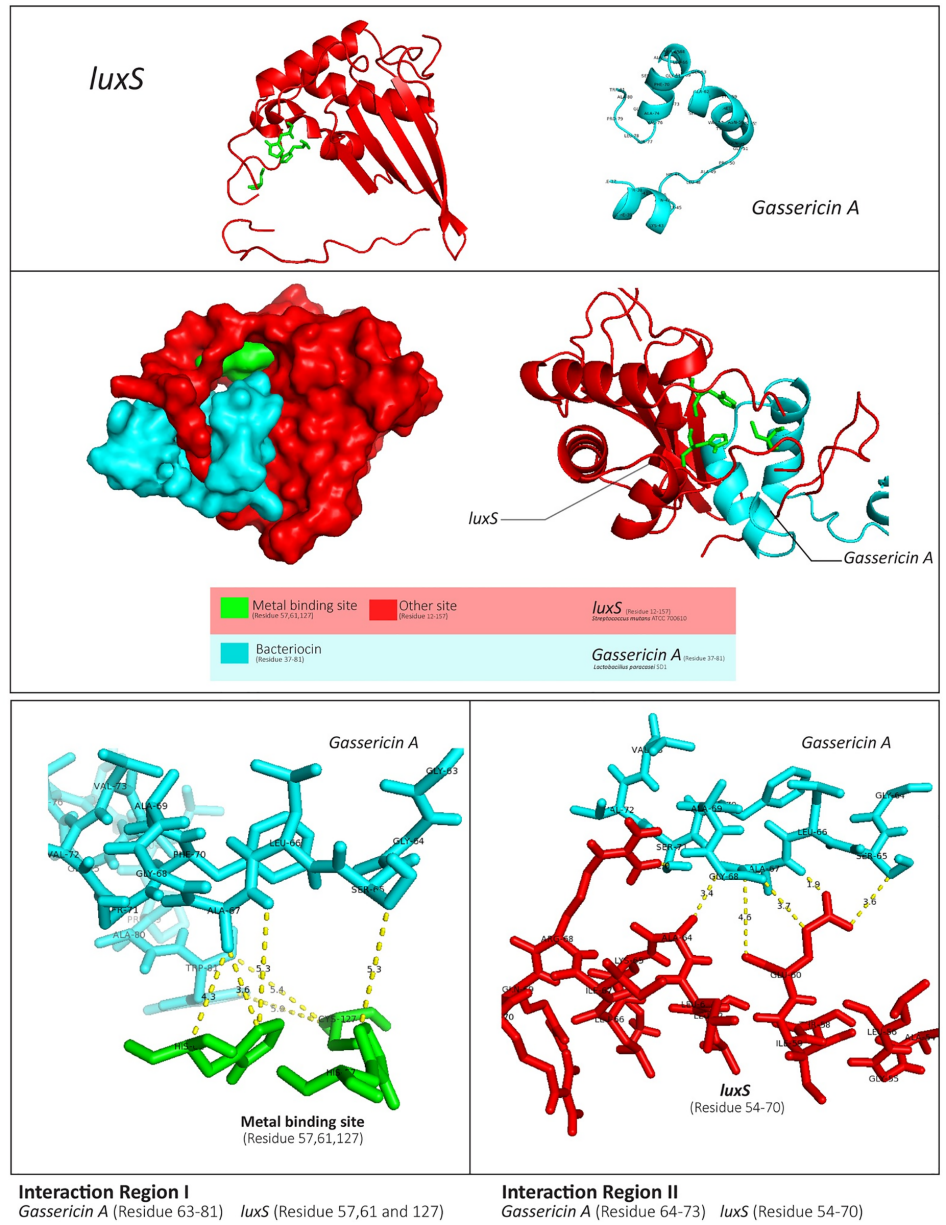
Protein-protein interaction between luxS and Gassericin A. The interaction between luxS (red) and Gassericin A (blue) was created using ClusPro and visualized by PyMOL. The minimum bond length between them in this area was approximately 3.60 Å at HIS-61 of luxS and ALA-67. The contact sites of luxS and Gassericin A were located at a metal binding site (green) and another site (red).

The results of docking from ClusPro are in S5 Table. The lowest energy scores among all the clusters for each interaction were selected to compare and illustrate the interactions. Interestingly, Gassericin A and luxS had the lowest energy scores for balanced, electrostatic, hydrophobic, and Van der Waals interactions; these scores were −1,396.2, −1,400.1, −2,055.9 and −142.1, respectively. The minimum bond length between them in this area was approximately 3.60 Å at histidine (HIS-61) of luxS and alanine (ALA-67), as shown in Fig 6. Their binding occurred at the metal binding site. Metal iron is required for luxS metabolism and stability. At this location in luxS (residues 57, 61, and 127), iron ion bound with these interfaces and made the enzyme stable. This *in silico* binding experiment showed that bacteriocin could strongly interfere in the binding of luxS with iron at position 61. In addition, the binding sites of luxS to bacteriocin at amino acid residues 12–157 could prevent binding between luxS and iron. Therefore, a lack of iron bound with luxS causes instability of the enzyme. Although the mode of action of Gassericin A was reported as membrane dissociation of the microorganism, this *in silico* binding result suggested an additional action of Gassericin A via luxS. However, the experiments are required to confirm this preliminary screening.

**Fig 7:**
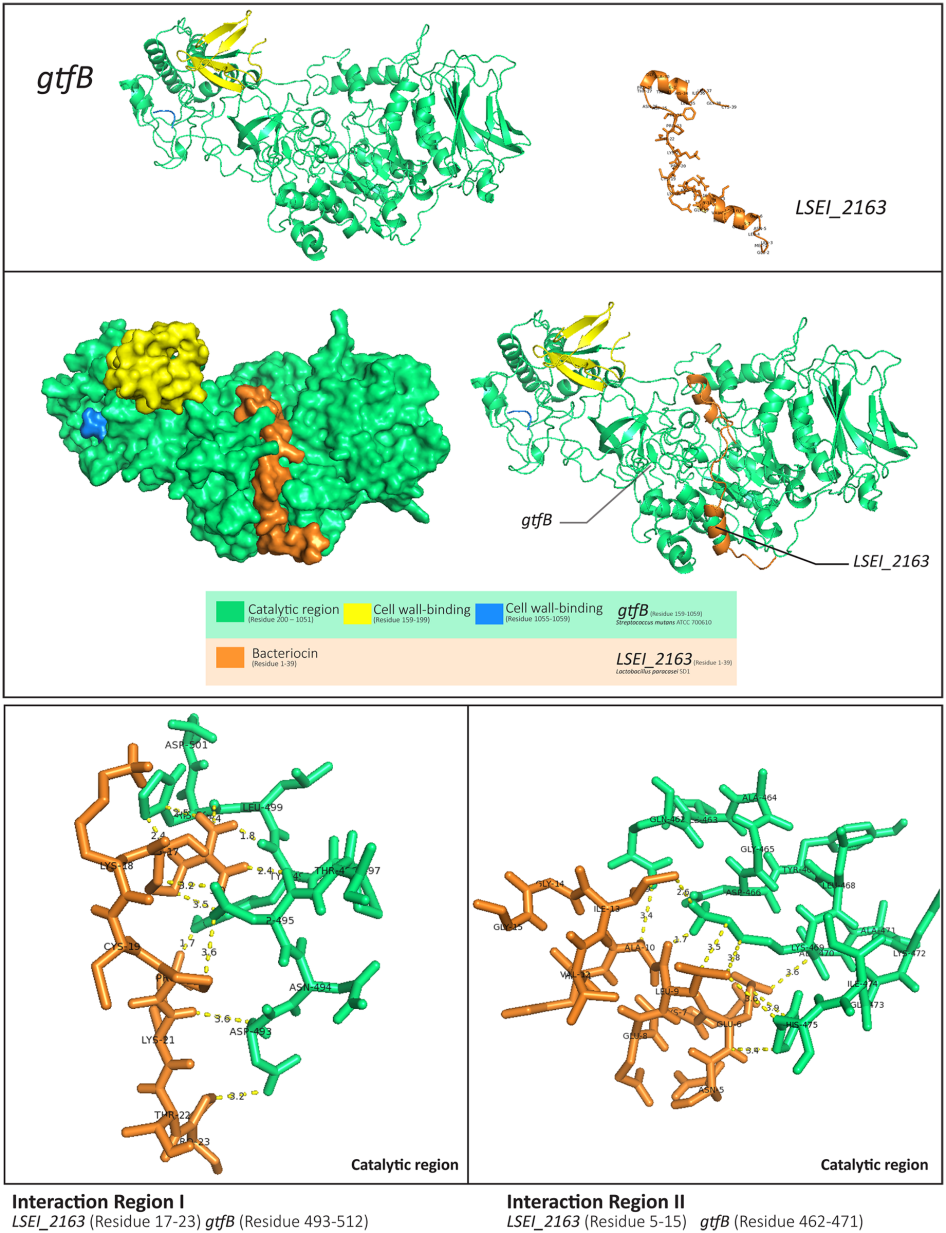
Protein-protein interaction between gtfB and LSEI_2163. The interaction between gtfB (green) and LSEI_2163 (orange) was created by using ClusPro and visualized by PyMOL. The minimum bond length between them was approximately 1.70 Å. The average bond length between them was 1.7–3.6 Å and the lowest energy of the balanced modes from ClusPro was around −1,000.

There were two interesting interactions between bacteriocins (LSEI_2163 and Gassericin A) and gtfB. They returned lower energy scores than LSEI_2386. The respective energy scores of the balanced, electrostatic, hydrophobic, and Van der Waals interactions were close in every mode, and we visualized them with PyMOL to view their binding sites. These two protein-protein interactions occurred at the different sites. Gassericin A’s interaction occurred at a cell wall-binding site, which may not affect the function of this protein. On the other hand, the result showed that LSEI_2163 bound to gtfB’s interface at the catalytic region, as shown in Fig 7. These interaction sites are located at residues (17–23) and (5–15) of LSEI_2163 and residues (493–512) and (446–487) of gtfB. The minimum bond length between them was approximately 1.70 Å. Bacteriocin could interfere with the production of insoluble glucans at the catalytic region of the enzyme. This analysis showed binding between bacteriocin and the target protein based on a computer model; therefore, further laboratory experiments to confirm the binding, such as yeast two hybrid experiments and investigation of required concentration to control the microorganism, are still required.

The results showed strong bonding between the bacteriocins and the target proteins of *S*. *mutans*, which supports putative antagonistic activity against *S*. *mutans* [18, 19] Therefore, the ab initio prediction of antibacterial activity by computational methods may be an alternative preliminary step before in vitro experimentation In conclusion, *L*. *paracasei* SD1 was not found to contain any suspicious genetic material indicating toxicity. Therefore, *L*. *paracasei* SD1 may be used as a probiotic in dairy products due to its ability to inhibit other bacterial strains.

## Supporting information

S1 Fig The bacterial gene and protein toxin identification workflows. The workflow was constructed with two main processes, a preliminary identification and the local blast and sequence alignment. The preliminary identification was implemented to search related gene and protein by using the BLASTN in the VFDB database and the BLASTX in the DBETH database. Then, the local blast and sequence alignment processes were used to check and confirm the similarity of the sequences of the identification.
(TIF)

S2 Fig Circular representation of the chromosome of *Lactobacillus paracasei* SD1 plasmids, plasmid (A) pSD1-1 and (B) pSD1-2. For both figures A and B, from the inner to outer circles: GC content, AT graph, CDS and Gene. The forward and reverse strand of CDS are yellow color and distinguished by using arrow direction. The ORFs are displayed in orange color.
(TIF)

S1 Table List of coding sequences identified in unique regions of the SD1. (DOCX)

S2 Table The protein toxin sequences in the SD1. The complete sequences of the Hemolysin, Hemolysin III, and lepB and molecular weight are given, respectively.
(DOCX)

S3 Table The report of antibiotic resistance proteins in the SD1. (DOCX)

S4 Table The bacteriocin sequences in the SD1 genome and plasmid. (DOCX)

S5 Table The lowest energy from *in silico* binding with the ClusPro between the bacteriocins of the SD1 and luxS and gtfB of *S*.*mutans*. (DOCX)
