# Supplementary figures and images for: In silico analysis of protein toxin and bacteriocins from Lactobacillus paracasei SD1 genome and available online databases
Source: PLoS One. 2017 Aug 24;12(8):e0183548. doi: 10.1371/journal.pone.0183548 (PMC5570283; doi:10.1371/journal.pone.0183548)

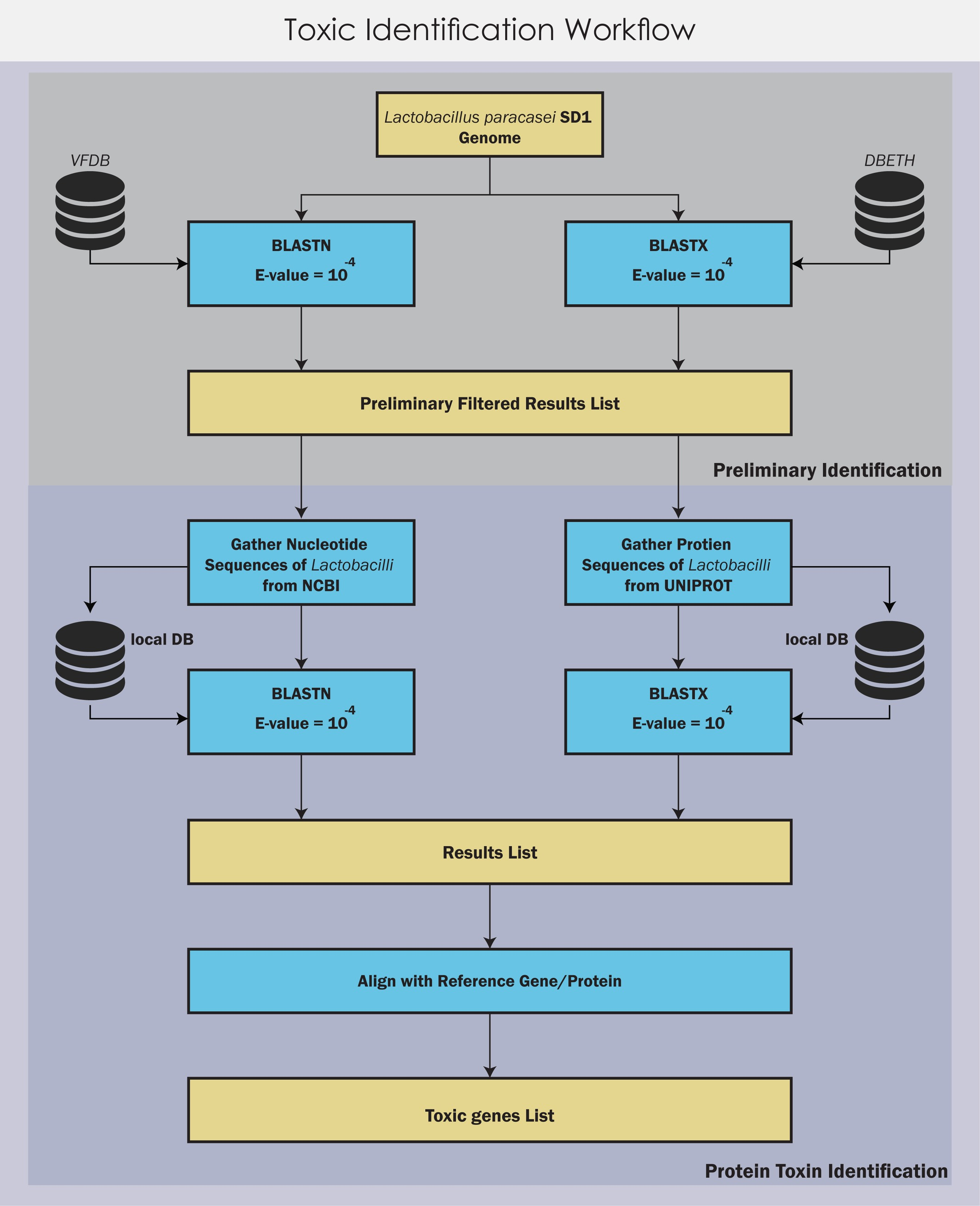

Supplement: S1 Fig — The workflow was constructed with two main processes, a preliminary identification and the local blast and sequence alignment. The preliminary identification was implemented to search related gene and protein by using the BLASTN in the VFDB database and the BLASTX in the DBETH database. Then, the local blast and sequence alignment processes were used to check and confirm the similarity of the sequences of the identification. (TIF) [file pone.0183548.s001.tif]

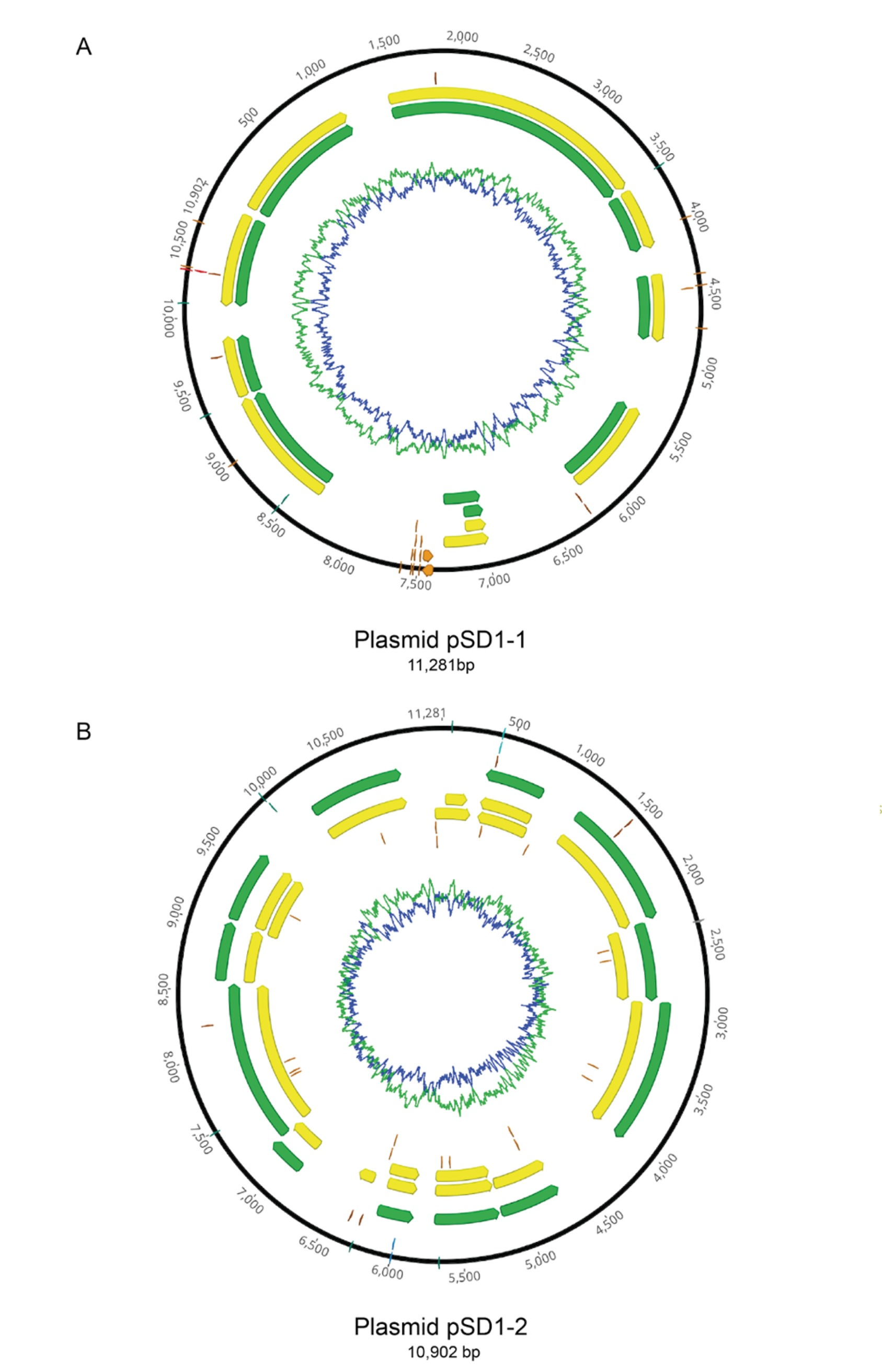

Supplement: S2 Fig — For both figures A and B, from the inner to outer circles: GC content, AT graph, CDS and Gene. The forward and reverse strand of CDS are yellow color and distinguished by using arrow direction. The ORFs are displayed in orange color. (TIF) [file pone.0183548.s002.tif]
