# Supplementary material for: In silico analysis of protein toxin and bacteriocins from Lactobacillus paracasei SD1 genome and available online databases
Source: PLoS One. 2017 Aug 24;12(8):e0183548. doi: 10.1371/journal.pone.0183548 (PMC5570283; doi:10.1371/journal.pone.0183548)
Supplement: S1 Table — (DOCX) [file pone.0183548.s003.docx]

**Table S1** List of coding sequences identified in the unique regions of the SD1 genome.

| **CDs** | **Length (bp)** | **Interval** |
| --- | --- | --- |
| Osmotically activated L-carnitine/choline ABC transporter, substrate-binding protein | 198 | 52,368 -> 52,171 |
| Osmotically activated L-carnitine/choline ABC transporter, substrate-binding protein | 762 | 53,099 -> 52,338 |
| Indole-3-glycerol phosphate synthase | 162 | 66,768 -> 66,607 |
| Mobile element protein | 921 | 78,363 -> 77,443 |
| PTS system, sucrose-specific IIB component | 1,992 | 104,459 -> 102,468 |
| Transcriptional regulator | 129 | 179,268 -> 179,140 |
| putative membrane protein | 501 | 212,592 -> 213,092 |
| Membrane protein | 360 | 213,153 -> 213,512 |
| Cell wall surface anchor family protein, FPXTG motif | 120 | 341,776 -> 341,895 |
| Mobile element protein | 1,278 | 1,771,223 -> 1,769,946 |
| Alpha-galactosidase | 162 | 1,803,210 -> 1,803,371 |
| Beta-lactamase class C and other penicillin binding proteins | 276 | 1,810,571 ->1,810,296 |
| Cell division transporter, ATP-binding protein FtsE | 300 | 1,941,287 -> 1,940,988 |
| Cell wall surface anchor family protein, FPXTG motif | 135 | 2,084,916 -> 2,084,782 |
| Alpha-galactosidase | 120 | 2,129,558 -> 2,129,439 |
| ABC transporter, ATP-binding protein | 174 | 2,222,904 -> 2,223,077 |
| oxidoreductase | 486 | 2,353,931 -> 2,353,446 |
| Sortase A, LPXTG specific CDS | 246 | 2,401,774 -> 2,401,529 |
| Acetyltransferase (isoleucine patch superfamily) | 126 | 2,435,062 -> 2,434,937 |
| UDP-glucose 4-epimerase | 129 | 2,496,726 -> 2,496,598 |
| Polysaccharide biosynthesis protein | 159 | 2,515,502 -> 2,515,344 |
