# Supplementary material for: In silico analysis of protein toxin and bacteriocins from Lactobacillus paracasei SD1 genome and available online databases
Source: PLoS One. 2017 Aug 24;12(8):e0183548. doi: 10.1371/journal.pone.0183548 (PMC5570283; doi:10.1371/journal.pone.0183548)
Supplement: S2 Table — The complete sequences of the Hemolysin, Hemolysin III, and lepB and molecular weight are given, respectively. (DOCX) [file pone.0183548.s004.docx]

**Table S2** Protein toxin sequences in the SD1.

| **Protein Name** | **Sequence** | **Molecular weight** (Da) |
| --- | --- | --- |
| Hemolysin | MGSDPDGQIWGQLILIVILTLINAGFAAAEIAVVSSSRTRMKAQADKGDRKAAKLVTIMKDSSNFLATIQVGITFAGFFASASAATTLADRVAPIFGGWSFAHEAAVILVTLILSYFSLVFGELYPKQVALQMTERVAKMSVTPISWLAKVMRPFVWLLSASTKLLMKLTPMEFNHEGETVTRDEMVSMIESGRNSGAIDPDEYQMFEGIISLSDTMAREVMVPRTDAFMVDAQEPDHTAIDAILNNIYSRIPVYEEDKDHVVGIVHIKNLLKEARRVGFDHVKIESVMTAPVFVPETITVDDLLTEMQVKQQQMAILLDEYGGVVGIVTIEDLLEEIVGEIDDESDQVEKLFTKQGDHDFVVSGRMPISDFNDLFKTDLDAPDVDTIAGYVLTQLGAIPSSHHSEKMQLAPGVLLATGKVEGSRLVNVHVHLSSEPEEASHEADS | 48,900 |
| Hemolysin III | MRVAKSKHYEFNNEMFSAITHAFALGLAVVGTIALGIKGANSGSQLELISYLGFGISLIILYTASTAFHGFYFSKARHVLQVLDHSGVFILIAGSYLPYCLVAIGGPLGIGLLIAIWALCFGGILYKLFFLNRFKHLETMIYVILGWLCLIGMVPLWHHLGPIGFWLLVAGGLAYTGGAMLYLQKGIPYIHVIWHLFVILGSLCMYISIYLFV | 23,481 |
| LepB | STLKTVLEFLVLFAVIFFASQLLMRYVLSKDVVQGTSMQPTLENGDRLYSIRVKKPKRNDIVVINAPDRPGSLYIKRVIGMPGDTVSSKDNQLSVNGKKIAEPYLNKKFATDEINKWASQQGLDASTIKFTNDFNIKTLSSTKSAKVPAGKYFVMGDNRLVSHDSR | 18,533 |
