# Supplementary material for: In silico analysis of protein toxin and bacteriocins from Lactobacillus paracasei SD1 genome and available online databases
Source: PLoS One. 2017 Aug 24;12(8):e0183548. doi: 10.1371/journal.pone.0183548 (PMC5570283; doi:10.1371/journal.pone.0183548)
Supplement: S3 Table — (DOCX) [file pone.0183548.s005.docx]

**Table S3** The report of antibiotic resistance proteins in the SD1.

|  | **Resfam Identifier** | |
| --- | --- | --- |
| **Antibiotic Mechanism** | ***Lactobacillus paracasei* SD1** | ***Lactobacillus paracasei* PSD1-1** |
| Acetyltransferase | RF0004 |  |
| ABC Transporter | RF0007, RF0088, RF0089, RF0107 | RF0007, RF089, RF0107 |
| Acetyltransferase | RF0005, RF0050 |  |
| Antibiotic Inactivation | RF0136 |  |
| Beta-Lactamase | RF0037, RF0053, RF0054, RF0055, RF0056, RF0057, RF0062, RF0064, RF0081, RF0087, RF0108, RF0119, RF0123, RF0125, RF0126, RF0161 |  |
| Gene Modulating Resistance | RF0035, RF0036, RF0042, RF0091, RF0106, RF0112, RF0114, RF0116, RF0117, RF0121, RF0154, RF0155 |  |
| Gylcopeptide Resistance | RF0149, RF0150, RF0151, RF0152, RF0153, RF0156, RF0160, RF0149, RF0150, RF0151, RF0152, RF0153, RF0156, RF0160 |  |
| MFS Transporter | RF0065, RF0104, RF0109, RF0127, RF0128, RF0129, RF0130, RF0131, RF0132, RF0134, RF0137 |  |
| Other Efflux | RF0022, RF0023, RF0051, RF0066 |  |
| Phosphotransferase | RF0033, RF0034, RF0052, RF0172, RF0173 |  |
| Quinolone Resistance | RF0074 |  |
| RND Antibiotic Efflux | RF0019, RF0097 |  |
| rRNA Methyltransferase | RF0067, RF0068, RF0069, RF0070, RF0071 |  |
| Target Protection | RF0133, RF0135 |  |
| **Total** | **80** | **3** |

*** Not found antibiotic resistance proteins in *Lactobacillus paracasei* plasmid pSD1-2
