# Supplementary material for: In silico analysis of protein toxin and bacteriocins from Lactobacillus paracasei SD1 genome and available online databases
Source: PLoS One. 2017 Aug 24;12(8):e0183548. doi: 10.1371/journal.pone.0183548 (PMC5570283; doi:10.1371/journal.pone.0183548)
Supplement: S4 Table — (DOCX) [file pone.0183548.s006.docx]

**Table S4** Bacteriocin sequences found in the SD1 genome and plasmid

| **Protein Name** | **Sequence** | **Molecular weight** (Da) | **Location** |
| --- | --- | --- | --- |
| LSEI_2386 | MKQFDEQKMVNMSDEELLGFIGGDSIRDVSPTFNKIRRWFDGLFK | 5,327 | genome |
| LSEI_2163 | MELLNEKELAHVIGGKRKCPKTPFDNTPGAWFAHLILGC | 4,336 | genome |
| Carnocin-CP52 immunity protein | MTDKRETLMSMLSKAYANPTIKAEPALRALIETNAKKVDEGDDDKAYVTAVTQLSHDISKYYLIHHAVPEELVAVFNYIKKDVPAADIDAARYRAQALAAGLVAIPIVWGH | 12,238 | genome |
| Enterocin Xβ | MCTMTNLEDKELSQITGGFAFVIPVAAILGFLASDAWSHADEIASGAKTGW | 5,373 | genome |
| Gassericin A | MLSTVEKLHMTKMEKTVLTIGSFVIAALMVTILFTNIYFIANKLGIHLAPGWYQDMVNYVSAGGSLAGAFSVVAGVTLPAW | 8,721 | plasmid |
