# Supplementary material for: In silico analysis of protein toxin and bacteriocins from Lactobacillus paracasei SD1 genome and available online databases
Source: PLoS One. 2017 Aug 24;12(8):e0183548. doi: 10.1371/journal.pone.0183548 (PMC5570283; doi:10.1371/journal.pone.0183548)
Supplement: S5 Table — (DOCX) [file pone.0183548.s007.docx]

**Table S5** The lowest binding energy from *in silico* analysis with the ClusPro between the bacteriocins of the SD1 and luxS and gtfB of *S.mutans*

| **Protein** | | **Lowest energy score in each mode** | | | |
| --- | --- | --- | --- | --- | --- |
| **Bacteriocins** | **Protein** **from**  *S.mutans* | **Balanced** | **Electrostatic-favored** | **Hydrophobic-favored** | **Van der Waals** |
| LSEI_2386 | luxS | -860.5 | -983.3 | -985.7 | -219.2 |
| LSEI_2163 | luxS | -1051 | -1,092.6 | -1,452.8 | -179.1 |
| Gassericin A | luxS | -1,396.2 | -1,400.1 | -2,055.9 | -142.1 |
| LSEI_2386 | gtfB | -719.6 | -794.0 | -1,047.8 | -220.9 |
| LSEI_2163 | gtfB | -1,076.8 | -1,128.7 | -1,200.4 | -203.6 |
| Gassericin A | gtfB | -925.1 | -1,042.7 | -1,417.8 | -162.1 |
